# Role of microRNA in Sex Steroid Hormones Signaling and Its Effect in Regulation of Endometrial, Ovarian, and Cervical Cancer: A Literature Review

**DOI:** 10.7759/cureus.54773

**Published:** 2024-02-23

**Authors:** Jina Fadl, Raghad A Aljuhani, Yusef H Albog, Ayda F Khraisat, Khlood A Alsubaie

**Affiliations:** 1 Obstetrics and Gynaecology, Batterjee Medical College, Jeddah, SAU

**Keywords:** ovarian cancer, cervical cancer, endometrial cancer, hormone therapy, micrornas

## Abstract

Worldwide, in 2020, an estimated 417,367 people were diagnosed with uterine cancer. Endometrial cancer accounts for more than 90% of all uterine cancers. The 15th most frequent cancer overall and the sixth most frequent cancer in women is endometrial cancer. Global ovarian cancer Incidence was diagnosed estimated at 313,959 new cases worldwide in 2020. Cervical cancer is the fourth most common malignancy in women worldwide. It has been demonstrated that sex steroid hormones (SSHs) have an essential role in regulating the susceptibility of cancer to cytotoxic therapy. Dysregulation of DNA repair contributes to genomic instability, aberrant cell survival, and cancer development as well as therapy resistance. Several crucial DNA repair components have been discovered to interact with the three main SSHs: androgen, estrogen, and progesterone. MicroRNA (miRNA) dysregulation has been associated with aberrant sex steroid hormone signaling as well as an increased risk of endometrial, cervical, and ovarian cancer. The expression of estrogen and progesterone receptors is modulated by a number of miRNAs, and it has been demonstrated that the miRNA expression profile may predict the way a patient would respond to hormone therapy. Additionally, particular miRNAs have been linked to the control of genes involved in signaling pathways connected to hormones. Recent research has shown that miRNAs can modify hormone signaling pathways and affect the expression of sex steroid hormone receptors.

Our goal in this literature review is to present an overview of current knowledge regarding the role of miRNAs in cancers regulated by sex steroid hormone pathways, as well as to identify particular miRNA targets for hormonal therapy.

## Introduction and background

MicroRNAs (miRNAs) control the expression of their target genes through mRNA degradation or translation suppression. These non-coding RNAs (ncRNAs) are proving to be crucial regulators in cellular processes and have been linked to the development of tumors. Modulating the miRNA activities may offer intriguing new possibilities for cancer therapy as our understanding of the target genes for miRNAs and the cellular behaviors that they affect grows [1]. The importance of ncRNAs including miRNAs and long non-coding RNAs (lncRNAs) in cancer diagnosis, prognosis, and therapy decisions is becoming recognized by researchers. These ncRNAs are important for controlling cellular metabolism and mutating cells into malignant ones. Recent research has revealed miRNAs to be promising therapeutic targets [2,3]. miRNAs can act as tumor suppressors or oncogenes [4]. The regulation of signaling pathways associated with cell proliferation, epithelial-mesenchymal transition, apoptosis, cell migration, and invasiveness by sexual steroid hormones functioning through their receptors is crucial for the growth of tumors. endometrial cancer, ovarian cancer, and cervical cancer are all caused by them [5,6]. The prognosis for patients with these tumors may be impacted by the presence of the estrogen receptor (ER), progesterone receptor (PR), and androgen receptor (AR), which are associated with clinicopathological factors. The progression and therapy of various cancers, including breast and endometrial cancer, are significantly influenced by the presence of AR, ER alpha, and PR [7]. Sex steroid hormones control the miRNA-mediated machinery of gene expression control in the cells of hormone-sensitive tissues in two ways that are currently understood: specifically by affecting the activity of individual miRNA molecules and non-specifically through altering the efficacy of miRNA biogenesis and the activity of the RNA-induced silencing complex. The biological effects of sex hormones in physiological settings are greatly improved by this downstream regulatory network. The regulatory effects of sex hormones are distorted by malignant transformation, which has a significant impact on the system of miRNA-regulated post-transcriptional regulation of gene expression [8].

The objectives of the study were (1) to determine the prevalence of AR, ER, and PR expression in endometrial cancer, ovarian cancer, and cervical cancer; (2) to clarify the relation to a cell proliferation indicator; and (3) to give an overview of the current understanding of the role of miRNAs in malignancies as well as to pinpoint specific miRNAs that are potential targets for hormone treatment.

## Review

Sex steroid hormones and miRNAs

Queirós et al. report that the expression and regulation of miRNA are done by estrogen via estrogen receptor beta (ERβ) [9]. Moreover, it’s demonstrated that the expression of miRNA is under the influence of steroid hormones, and any disturbance in the enzyme generating the mature miRNA (Dicer1) leads to developmental and functional abnormalities in the female reproductive tract [10]. Metastasis of endometrial cancer is increased with downregulation of miRNA-449a. Moreover, the migration and invasion of endometrial cancer are significantly inhibited by miR-449a. The inhibitory effect of miRNA-449a on endometrial cancer metastasis is done by downregulating the steroid receptor coactivator (SRC) [11]. In addition, the migration of ovarian cancer cells is shown to be suppressed by miRNA-200 cluster overexpression [12]. In cervical cancer, the presence of lymph node metastasis (LNM), an increase in tumor size, advanced stage, and histological grade are associated with up-regulated expression of miR-20a, so miR-20a has a significant role in the progression and metastasis of cervical cancer [13].

Role of sex steroid hormone in endometrial cancer

The sixth most commonly occurring female cancer worldwide is endometrial cancer, which accounts for more than 90% of the cases of uterine tumors. It arises from the endometrium and is divided into subgroups, including serous, clear cell, endometrioid, mixed cell adenocarcinoma, and other rare types [14,15]. Endometrial cancer has several causes and risk factors, but the main focus of this literature review is on sex steroid hormones. This type of cancer is frequently a hormonally sensitive disease due to overstimulation by estrogen to the endometrium [16]. Endometrial hyperplasia and adenocarcinoma may occur as a result of unopposed estrogen action caused by insufficient progesterone that leads to overexposure to estrogen [17]. Progesterone, on the other hand, functions as an antagonist, preventing cell division, reducing ER, and encouraging cell differentiation [18].

Role of sex steroid hormone in ovarian cancer

Among women, the seventh most common cancer is ovarian cancer. It has different types of origin, including epithelial (the most common) and non-epithelial [19]. Ovarian carcinogenesis is thought to be influenced by steroid hormones, particularly estrogen and progesterone. Increased progesterone exposure and availability are typically protective against ovarian cancer (opposing tumorigenesis), given that it has reduced ovarian cancer cells' invasion, migration, and proliferation [20]. The development of ovarian cancer is highly linked to the estrogen hormone, as it is suggested that it has a relationship with phosphatase and tensin homolog (PTEN), which is a tumor suppressant that regulates migration, cell proliferation, and survival. Estrogen can reduce and activate PTEN protein expression via the genomic pathway and the non-genomic pathway, which promotes ovarian cancer migration and proliferation [21].

Role of sex steroid hormone in cervical cancer

There’s a clear relationship between sex steroid hormones (specifically estrogen and progesterone) and cervical cancer. Firstly, the immunosuppressive properties and potential link to human papillomavirus (HPV) infection make progesterone the main probable hormone in cervical cancer. Cells with PRs tend to get infected by HPV. An increase in HPV mRNA and viral replication is greatly stimulated by progesterone and glucocorticoid hormones. On the other hand, estrogen has been demonstrated to decrease the risk of developing a primary HPV infection; however, this effect may be insignificant if the virus has already been implemented. Where cervical neoplasia started and formed, estradiol underwent 16-alpha-hydroxylation, leading to the formation of 16-alpha-hydroxyestrone, which is connected to the malignant transformation of HPV-transfected estrogen-sensitive cells. High serum estrogen levels may improve outcomes once invasive cancer has been diagnosed; however, high serum progesterone levels have been linked to poor outcomes [22].

Association between miRNA and endometrial cancer

Endometrial cancer, a prevalent form of cancer, is projected to impact 10-20 out of every 100,000 women worldwide, and its incidence continues to rise. Surgery is the primary treatment for early-stage endometrial cancer patients. However, advanced endometrial cancer carries a grim prognosis, with a five-year overall survival rate of approximately 15-17% [23]. While a few molecular classification systems and biomarkers have been proposed as supplementary tools to current risk stratification methods, none of them have gained widespread usage or become established as standard practices in clinical settings. Consequently, a key clinical challenge remains the identification of efficient and accurate biomarkers for early diagnosis and prognosis of endometrial cancer [24]. In order to develop effective targeted treatments, it is crucial to investigate new biomarkers [23].

miRNAs, a specific type of short non-coding RNAs measuring 19-24 nucleotides in length, have been found to regulate gene expression by binding to specific mRNAs through antisense complementarity or complementarity. Numerous studies have demonstrated that several miRNAs display abnormal expression in various types of tumors and act as oncogenes or tumor suppressor genes, contributing to carcinogenesis and disease progression. MiRNA expression has been linked to cell growth, metastasis, invasion, and response to therapy. Furthermore, miRNAs are highly sensitive, specific, and stable molecules, making them excellent markers for identifying specific tumors and monitoring their development. The advent of various miRNA sequencing methods, particularly next-generation sequencing technology, has enabled the identification of miRNA signatures in endometrial cancer. These signatures have the potential to distinguish endometrial cancer from normal tissues and provide insights into the molecular diagnosis and prognosis of the disease. Previous research has explored the relationship between endometrial cancer and miRNAs, revealing potential non-invasive biomarkers such as has-mir337p, let-7b, and miR135a. Abnormal miRNA expression patterns have also been strongly associated with the early stages of carcinogenesis. The upregulation of several oncogenic proteins, including ERBB2, EGFR, EPHA2, BAX, GNA12, GNA13, and JUN, suggests that miRNA expression patterns in endometrial cancer are correlated with their respective target mRNAs. This finding further supports the significant role of miRNA-target mRNA interactions in carcinogenesis. Therefore, miRNAs hold promise as novel non-invasive biomarkers to diagnose and make prognosis of endometrial cancer, guiding surgical interventions, and advancing our understanding of the development of endometrial cancer [24].

Association between miRNA and ovarian cancer

Ovarian cancer is a leading cause of death among gynecological malignancies, with 295,414 new cases reported worldwide in 2018. The early development of distant metastasis contributes to the low five-year survival rate of less than 45%. Current biomarkers used in clinical practice, such as CA125, lack specificity, and early detection is challenging with ultrasound exams. Therefore, it is crucial to reevaluate newly discovered ovarian cancer biomarkers and understand their associations with important molecules. This is essential for developing strategies for the prevention and management of ovarian cancer [25]. In recent years, the use of expression profiles and bioinformatics analysis has generated large amounts of omics data. Some studies in the past three years have utilized expression profiles and bioinformatics approaches to identify significant genes in ovarian cancer. However, many of these studies used similar microarray profiles, which may have led to lower accuracy and false-positive results. 

miRNAs are non-coding RNAs that bind to complementary regions in mRNA, resulting in negative regulation of downstream gene expression and mRNA silencing. Abnormalities in miRNAs have been observed in various cancers, including ovarian cancer, where they can alter the expression of target genes. Several studies have explored the potential of miRNA expression profiles as molecular markers for ovarian cancer [25]. Numerous miRNAs have been identified to play a role in the regulation of various cellular processes associated with ovarian cancer, based on their functions in cancer treatment. For example, studies have highlighted the role of exosomal miR-205 in tumor angiogenesis and identified miR-21 as a regulator of macrophage polarization, which increases chemoresistance in ovarian cancer cells. MiR-582-3p has been found to influence the growth, differentiation, and survival of tumor cells and also plays a role in tumor invasion, vascular infiltration, and metastasis. However, there is limited research specifically addressing the function and expression of miR-582-3p in ovarian cancer development [26].

Association between miRNA and cervical cancer

Cervical cancer is prevalent cancer that affects women worldwide. According to data from the Global Cancer Observatory database, there were approximately 570,000 new cases of cervical cancer in 2018. China has the highest incidence rate, with 106,430 cases reported. Despite advancements in therapeutic options such as surgery, radiotherapy, and chemotherapy, a significant number of patients still experience relapse or metastasis, leading to a high mortality-to-incidence ratio of around 30-50%. Therefore, identifying patients at high risk of poor prognosis early on is a significant challenge for gynecologists [27]. Growing evidence suggests that the advanced International Federation of Gynecology and Obstetrics (FIGO) stage is associated with a higher risk of recurrence and shorter five-year survival. The FIGO staging system is widely accepted as a predictive indicator for cervical cancer in clinical settings. However, some studies indicate that the FIGO staging system's predictive performance could be improved, as survival variations have been observed even within the same stage. Consequently, there is a research focus on finding more accurate prognostic biomarkers. Recent advancements in sequencing technology and bioinformatics have led to the identification of molecular biomarkers that can predict survival in cervical cancer patients more accurately than in the FIGO staging system. For example, one study identified a prognostic signature composed of five messenger RNAs (mRNAs) that encode proteins (ITM2A, DSG2, SPP1, EFNA1, and MMP1), which were found to be independent indicators for survival prediction in cervical cancer patients. Another study discovered a five-mRNA signature (GALNTL6, ARSE, DPAGT1, GANAB, and FURIN) that could predict disease-free survival [27].

MiRNAs, which can be found either in a free form in the bloodstream or integrated into exosomes (membrane vesicles released by various cell types, including tumor cells), play a role in intercellular communication and genetic exchange between cells. Exosomal miRNAs have gained attention as potential cancer biomarkers due to their stability and detectability in biological fluids. They have been found to influence the formation and spread of cervical malignancies, along with other non-coding RNAs, tumor suppressors, and oncogenes. Previous research has identified several cancer-specific miRNAs that can be used for early detection of cervical cancer. However, there is limited research on cervical cancer and exosomal miRNAs. Alterations in miRNA expression profiles have been reported in cervical cancer, and studies have shown that miRNAs can modulate the expression levels in cervical cells infected with HPV. Exosomes are also implicated in various processes related to cancer, including metastasis, angiogenesis, tumor immunity, and treatment resistance. While miRNAs have been established as diagnostic biomarkers for cervical cancer and precancerous lesions, further investigation is needed to understand the role of exosomal cervical cancer-derived miRNAs as diagnostic biomarkers and their contribution to cervical cancer progression [28].

Regulation of sex steroid hormone receptors expression and hormone signaling pathway through miRNA in endometrial, ovarian, and cervical cancer

**Figure 1:**
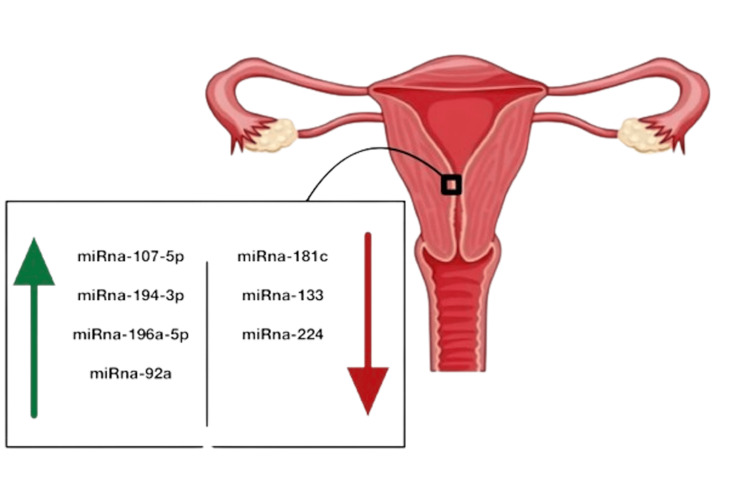
miRNA control and regulation of endometrial tissue in cases of endometrial cancer The green arrow indicates miRNA that promotes tumor growth The red arrow indicates miRNA that down-regulates tumor growth The figure was created by Raghad A. Aljuhani

In endometrial cancer, it has been shown that a variety of miRNAs regulate the production of estrogen and PRs in endometrial cells (Figure 1) [29]. Targeting ER, miRNA-107-5p promotes tumor growth and invasion, whereas miRNA-194-3p and miRNA-196a regulate the synthesis of the PR-A and PR-B proteins, respectively [30,31]. Additionally, miR-92a promotes epithelial endometrial cell proliferation and progesterone resistance, whereas miRNA-181c inhibits estrogen-dependent endometrial cancer cell development via targeting PTEN [30,32]. However, the exact mechanisms through which miRNAs control ER in the endometrium are still not completely known [30]. The downregulation of miRNA-133 and miRNA-224 appears to be a molecular marker of endometrial cancer [30].

**Figure 2:**
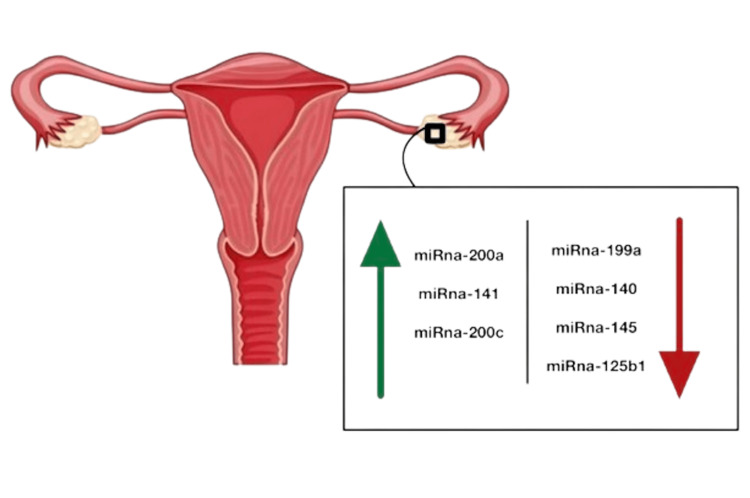
miRNA control and regulation of ovarian tissues in ovarian cancers The green arrow indicates miRNA which is over-expressed in ovarian cancers The red arrow indicates miRNA which is down-regulated in ovarian cancer The figure was created by Raghad A. Aljuhani

The most fatal gynecological cancer is ovarian cancer. Recent research has assessed miRNAs in ovarian cancer compared to normal tissues to find miRNAs that vary in expression [33,34]. The regulation of mammalian reproduction is regulated by miRNAs, which are expressed in the ovary. Timoneda et al. systematically studied the expression of porcine miRNAs and discovered that the ovary preferentially expresses let7a, miR-25, and miR-106a [35]. A deep sequencing technique was used to analyze the miRNA transcriptome in the mature ovary and testis of pigs. The top three unique miRNAs for the ovary and testis, according to their findings, are miR-21-5p, miR-143-3p, and members of the let-7 family. They also serve cellular housekeeping roles during ovarian and testicular development. As a result, miR-10b, miR-26a, miR-21, miR-140, and miR-101 are upregulated in the ovary rather than in the testis. MiR-378, miR-1, miR-206, miR-379, miR-127, and miR-411 are all downregulated in the ovary relative to the testis [36]. They were the first to analyze genome-wide miRNA expression patterns from both normal ovary tissues and ovarian cancer tissues, and they discovered that miRNA expression differed between the two groups. Ovarian cancer overexpressed the miRNAs miR-200a, miR-141, miR-200c, and miR-200b, while the most downregulated miRNAs were miR199a, miR-140, miR-145, and miR-125b1. These four downregulated miRNAs shared BRCA1-associated protein (BAP1) as an oncosuppressor target (Figure 2) [37].

**Figure 3:**
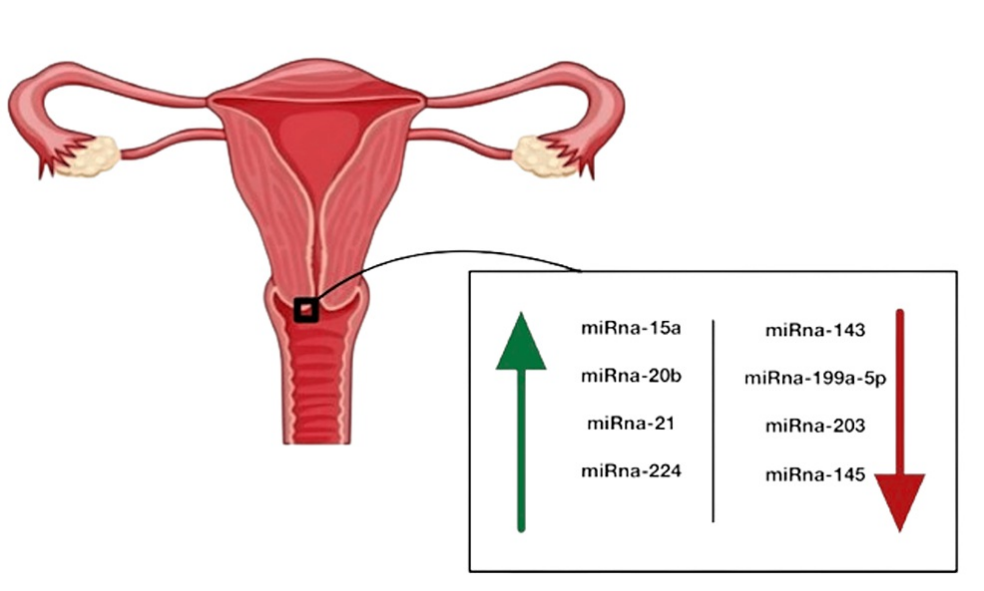
miRNA control and regulation of cervical tissues in cases of cervical cancers The green arrow indicates miRNA increased in cervical cancer patients The red arrow indicates miRNA which is sharply reduced in cervical cancer patients The figure was created by Raghad A. Aljuhani

In cervical cancer, one of the most commonly diagnosed tumors and the third leading cause of cancer death in women is cervical cancer [38]. By affecting their target genes, miRNAs play a critical role in the initiation, growth, and metastasis of cancer. Understanding the connections between target genes and miRNAs is necessary to understand the regulatory mechanisms of miRNAs in tumor development, metastasis, and invasion [39]. In cervical cancer tissues, 29 miRNAs showed differential expression, with 13 miRNAs up-regulated and 16 miRNAs down-regulated [40]. Although microRNA (miR) 130a3p expression levels were higher in cervical cancer tissues compared to healthy tissues, ER and AR expression decreased sequentially from healthy cervical tissues to cervical intraepithelial neoplasia tissues to cervical cancer tissues. By specifically targeting ER and AR, miR-130a-3p promoted cervical cancer cell invasion and proliferation [41]. According to previous studies on cervical cancer, miR-15a, miR-20b, miR-21, and miR-224 are obviously increased in cervical cancer tissues, and miR-143, miR-199a-5p, miR-203, and miR-145 are sharply reduced (Figure 3) [42].

miRNA targets hormone therapy in endometrial, ovarian, and cervical cancer

MiRNAs are single biomarkers that can be used to diagnose and treat a variety of gynecological malignancies. They can be used to distinguish between different types of cancer [42]. In endometrial, ovarian, and cervical cancers, abnormal miRNA expression is crucial for the formation and progression of tumors [43].

Previous studies demonstrated that MiR-206 was one of the first discovered [44,45,46,47]. MiR-221 and -222 have also been demonstrated to directly target ER [47,48]. In addition, miR-22 was demonstrated to cause inhibition of ERα target gene transcripts [47,49]. Another example of a miR that targets downstream regulators of hormone signaling is steroid-regulated miR-27a: ZBTB10 and Prohibitin, two hormone receptor corepressors, are both suppressed by miR-27a. MiR-27a also indirectly activates ER since Sp1, which is essential for ER production and the E2 response, is derepressed by miR-27a. A single nucleotide variation in miR-27a's terminal loop, which is projected to prevent pre-miR-27a processing, is related to a lower risk of familial premenopausal breast cancer, which is interesting and consistent with these findings. It is yet unknown how this mutation will affect miR-27a's capacity to suppress ZBTB10 which will indirectly increase ER activity [50-52]. Interestingly, it has also been shown that miR-219, miR-373,miR-302c, miR-301, miR-372, miR-193b, miR-130a/b, miR-93 target ER and have been provided reduced ER activity [47].

As demonstrated, other miRs also directly target AR. demonstrated that ectopic over-expression of miR-205 dramatically reduced the levels and activity of AR protein [53]. Similar to miR-185, miR-34's ectopic expression resulted in lower AR levels [54-56]. Interesting in this context is the down-regulation of AR also caused by miR-135b, miR-297, miR-299-3b, miR-421, miR-499a/b, miR-634, miR-9, miR-654-5b, miR-124, and miR-27a [54].

According to prediction software tools, the higher levels of the miRNAs (miR-1-3p, miR-122-5p, miR-125b-5p, miR-145-5p, and miR-150-5p) detected in endometriotic tissue and/or fluids are projected to target PRs [57-64]. Unfortunately, no studies have examined their role in endometrial or endometriotic tissues and/or cells or their capacity to control PR expression in any tissues. Studies in cancer biology done in vitro can provide information on the possible role of these miRNAs [57,58]. miRNAs known to target ER, AR, and PR are summarized in Table 1.

**Table 1:** Targets and status of specific miRNAs to estrogen receptor (ER), androgen receptor (AR), and progesterone receptor (PR) ERα for estrogen receptor alpha PR for progesterone receptors AR for androgen receptors ↑ indicates an increase in the number of receptors as a result of corresponding miR ↓ indicates a decrease in the number of receptors as a result of corresponding miR

miRNAs	Target receptor	Direction of activity	Reference
miR-206	ERα	↓	Iorio et al. [44], Adams et al. [45], Kondo et al. [46], and Leivonen et al. [47]
miR-121/222	ERα	↓	Zhao et al. [48] and Leivonen et al. [47]
miR-22	ERα	↓	Leivonen et al. [47] and Pandey et al. [49]
miR-27a	ERα	↑	Mertens-Talcott et al. [50] and Li et al. [51]
miR-219	ERα	↓	Leivonen et al. [47]
miR-373	ERα	↓	Leivonen et al. [47]
miR-302c	ERα	↓	Leivonen et al. [47]
miR-301	ERα	↓	Leivonen et al. [47]
miR-372	ERα	↓	Leivonen et al. [47]
miR-193b	ERα	↓	Leivonen et al. [47]
miR-130a/b	ERα	↓	Leivonen et al. [47]
miR-93	ERα	↓	Leivonen et al. [47]
miR-205	AR	↓	Hagman et al. [53]
miR-185	AR	↓/↓	Östling et al. [54] and Qu et al. [55]
miR-34	AR	↓/↓	Östling et al. [54] and Kashat et al. [56]
miR-135b	AR	↓	Östling et al. [54]
miR-297	AR	↓	Östling et al. [54]
miR-299-3b	AR	↓	Östling et al. [54]
miR-421	AR	↓	Östling et al. [54]
miR-449a/b	AR	↓	Östling et al. [54]
miR-634	AR	↓	Östling et al. [54]
miR-9	AR	↓	Östling et al. [54]
miR-654-5p	AR	↓	Östling et al. [54]
miR-124	AR	↓	Shi et al. [65]
miR-27a	AR	↑	Fletcher et al. [66]
miR-1-3p	PR	↓	Teague et al. [57] and Filigheddu et al. [58]
miR-122-5	PR	↓	Wang et al. [59] and Maged et al. [60]
miR-125b-5	PR	↓	Filigheddu et al. [58], Yang et al. [61], Vanhie et al. [62], Moustafa et al. [63]
miR-145-5	PR	↓	Teague et al. [57], Filigheddu et al. [58], Cosar et al. [64]
miR-150-5	PR	↓	Cosar et al. [64] and Moustafa et al. [63]

## Conclusions

Sex steroid hormones acting through their receptors are essential for the control of signaling pathways involved in cell proliferation, epithelial-mesenchymal transition, apoptosis, cell migration, and invasiveness. They contribute to the development of endometrial cancer, ovarian cancer (OC), and cervical cancer (CC). The presence of the estrogen receptor (ER), progesterone receptor (PR), and androgen receptor, which are linked to clinicopathological variables, may affect the prognosis among those with these cancers. Steroid hormones have been demonstrated to affect the production of microRNAs, and any alterations in the enzyme responsible for producing mature microRNAs may have effects on hormone signaling. Numerous microRNAs have been shown to control the manufacture of ERs and PRs in endometrial cells in endometrial carcinoma. By specifically targeting ERs, miR-107-5p encourages tumor development and invasion, whereas miR-194-3p and miR-196a control the production of PR proteins. The most lethal form of gynecological cancer is OC, and recent studies have examined how microRNA expression differs in OC and healthy tissues. The three most distinctive microRNAs found in the ovary and testis are miR-21-5p, miR-143-3p, and let-7 family members. One of the most regularly viewed tumors and a major contributor to cancer-related deaths in women is CC. MiR-15a, miR-20b, miR-21, and miR-224 have all been shown to significantly increase in CC tissues, whereas miR-143, miR-199a-5p, miR-203, and miR-145 have been found to significantly decrease.
